# Patterns of Gene Flow between Crop and Wild Carrot, *Daucus carota* (Apiaceae) in the United States

**DOI:** 10.1371/journal.pone.0161971

**Published:** 2016-09-07

**Authors:** Jennifer R. Mandel, Adam J. Ramsey, Massimo Iorizzo, Philipp W. Simon

**Affiliations:** 1 Department of Biological Sciences, The University of Memphis, Memphis, Tennessee, United States of America; 2 W. Harry Feinstone Center for Genomic Research, The University of Memphis, Memphis, Tennessee, United States of America; 3 Plants for Human Health Institute, Department of Horticultural Science, North Carolina State University, Kannapolis, North Carolina, United States of America; 4 USDA-Agricultural Research Service, Vegetable Crops Unit, University of Wisconsin-Madison, Wisconsin, United States of America; Chinese Academy of Sciences, CHINA

## Abstract

Studies of gene flow between crops and their wild relatives have implications for both management practices for cultivation and understanding the risk of transgene escape. These types of studies may also yield insight into population dynamics and the evolutionary consequences of gene flow for wild relatives of crop species. Moreover, the comparison of genetic markers with different modes of inheritance, or transmission, such as those of the nuclear and chloroplast genomes, can inform the relative risk of transgene escape via pollen versus seed. Here we investigate patterns of gene flow between crop and wild carrot, *Daucus carota* (Apiaceae) in two regions of the United States. We employed 15 nuclear simple sequence repeat (SSR) markers and one polymorphic chloroplast marker. Further, we utilized both conventional population genetic metrics along with Shannon diversity indices as the latter have been proposed to be more sensitive to allele frequency changes and differentiation. We found that populations in both regions that were proximal to crop fields showed lower levels of differentiation to the crops than populations that were located farther away. We also found that Shannon measures were more sensitive to differences in both genetic diversity and differentiation in our study. Finally, we found indirect evidence of paternal transmission of chloroplast DNA and accompanying lower than expected levels of chloroplast genetic structure amongst populations as might be expected if chloroplast DNA genes flow through both seed and pollen. Our findings of substantial gene flow for both nuclear and chloroplast markers demonstrate the efficiency of both pollen and seed to transfer genetic information amongst populations of carrot.

## Introduction

Cultivated plant species and their sexually-compatible wild relatives often overlap in terms of geographic proximity and phenology [1]. This overlap provides the opportunity for gene flow between crops and their wild relatives [2,3]. Farmers and breeders are often concerned with the potential for wild allelic contamination *into* agricultural fields, which can hinder production efficiency. While, natural land managers may wish to minimize gene flow *out* of agricultural fields into natural, often weedy, populations of closely related species. Moreover, given a genetically modified (GM; a.k.a. genetically engineered) crop system, an additional consideration is the risk of escape of transgenes from an agricultural setting to the wild by gene flow through both hybridization, and/or introgression [4–7].

Gene flow between non-GM crops and their wild relatives has the potential to increase weediness of wild populations (see examples in relatives of canola, millet, radish rice, rye, sugar beet, and sorghum [8–11]). Moreover, increases in invasiveness and accompanying range expansions of a wild species as a result of introgression with crop relatives has been investigated in a number of species [12–17]. Given the growing knowledge that escaped transgenes, especially those conferring insect or pathogen resistance, could have detrimental effects on natural communities, there has been much interest in understanding patterns of gene flow between crops and their wild relatives [18–22]. Moreover, the recent announcement that genetically engineered crops are not harmful to human health combined with rising interest in genome editing technologies, could lead to an increase in the production of engineered crops in the near future and a greater focus on the potential for gene escape [23].

One particularly weedy crop wild relative is wild carrot, *Daucus carota* subsp. *carota* L., (a.k.a. Queen Anne’s Lace). Wild carrot is abundant in temperate regions across the globe and is widely distributed across much of the United States where it is often found along roadsides, abandoned fields, and pastures. Wild carrot is the progenitor of the cultivated carrot, *D*. *carota* subsp. *sativus*, and the two subspecies are sexually compatible [24–26]. The cultivated carrot was likely domesticated in Central Asia roughly 1,100 yr ago [27,28] and is grown worldwide from both open pollinated and hybrid seed. Currently, no genetically engineered carrot products are available though much work has been carried out, see [29]; however, the carrot system is a good model for studying patterns of gene flow between crop-wild complexes. In many carrot-producing regions throughout the world, wild carrot populations can be found growing in close proximity to cultivated carrot fields [30,31]. Observations of wild carrot growing *within* cultivated carrot fields have also been reported [25,26,32]. In the eastern, midwestern, and northwestern United States, Queen Anne’s Lace populations and crop carrot fields often occur in close proximity where gene flow between wild and crop populations of carrot is likely. Given the concerns for gene flow in both directions, we sought to investigate levels of crop-wild gene flow in two regions of carrot production within the United States utilizing population genetic methods.

In plants, gene flow among populations can occur through both pollen and seed owing to the differential transmission of nuclear and organellar genomes [33–36]. Nuclear genomes are biparentally inherited; thus, genetic material moves through both seed and pollen. Organellar genomes (mitochondrial and chloroplast) however, usually only move via pollen or seed because of uniparental inheritance. In flowering plants, uniparental inheritance is typically maternal for both the mitochondrial and chloroplast genomes. This differential mode of inheritance can lead to varying levels of gene flow for pollen versus seed since pollen generally moves greater distances than seed. In an extensive review, Petit et al. [37] found that when compared to biparentally inherited genomes, those that are maternally inherited experience more population subdivision—usually an order of magnitude less in terms of gene flow. Cytoplasmic male sterility is a maternally-inherited trait that is widely used in carrot breeding with molecular marker evidence supporting a maternal inheritance model, but with relatively few markers [31,38]. Similarly, maternal inheritance was observed for a plastid polymorphism [39], but few plastid molecular markers have been evaluated in carrot. So for both organellar genomes, predominant, but not exclusive, maternal inheritance is documented. However, previous work on a limited sample of wild carrot populations in the eastern United States demonstrated lower than expected levels of population sub-structuring of mitochondrial genomes (i.e., indicating the potential for greater gene flow than expected for organellar genes) [40]. We therefore wished to compare the potential for seed and pollen to differentially contribute to gene flow in the current study by comparing nuclear and cytoplasmic (chloroplast) gene markers.

Given strict uniparental inheritance of organelles (maternal in the case of angiosperms), the comparison of gene flow in seed versus pollen is fairly straightforward. However, it is becoming increasingly evident that the rule of maternal inheritance in flowering plants is not always the case (see *Tansley Review* by [41]). In fact, in most taxonomic groups where this has been examined, some degree of non-strict-maternal inheritance, often termed *paternal leakage*, has been observed. From fish to fungi, natural and experimental studies have demonstrated DNA in the offspring originating from the paternal donor (reviewed recently in [42]). In wild carrot, indirect evidence of paternal leakage has been demonstrated through the observation of offspring that are heteroplasmic containing a mixture of at least two types of mitochondrial or chloroplast genomes [40,43]. Paternal leakage of organellar genomes should lead to less population subdivision than would typically be expected since organellar genes would move through both seed and pollen. Values of population sub-structuring (e.g., F_ST_ measures) would tend to approach those of nuclear genomes depending on the degree of paternal leakage. Moreover, because the two genomes (mitochondrial and chloroplast) need not leak together, values of population subdivision may be different for gene markers of the two genomes [41]. Bearing this in mind, we also wished to evaluate the results of gene flow in nuclear and chloroplast genomes in the context of any potential paternal leakage that might occur between crop and wild carrot populations.

Recently several authors have suggested additional methods (beyond F_ST_ and related measures) for assessing gene flow among plant populations [44–46] and in particular between crop and wild species populations [47]. These authors suggest using metrics, such as those based on Shannon information theory (i.e., from Ecology: Shannon diversity indices) [48], to investigate allele frequency variation within and among populations. Such indices have traditionally been employed in ecological studies to evaluate species diversity and evenness. One benefit of using Shannon diversity indices is that these measures account for allele identity along with frequency. Moreover, Campbell et al. [47] suggest comparing both total and multilocus measures when assessing the potential for gene flow between crop and wild populations. We therefore calculate both traditional population genetic metrics and ecological Shannon diversity indices and make comparisons at both the total and multilocus levels in the present study.

The overall goals of this study are to 1) investigate patterns of gene flow between crop and wild carrot across two regions of the United States using both nuclear and chloroplast DNA markers, 2) investigate evidence for chloroplast heteroplasmy in samples of both crop and wild carrot, and 3) compare conventional population genetic metrics of gene flow and genetic diversity to those that make use of species diversity indices of Shannon information.

## Materials and Methods

### Plant sampling strategy and genomic DNA isolation

The sampling strategy for wild carrot consisted of collecting leaf material or seeds (subsequently grown in the University of Memphis Greenhouses to obtain leaf material) from populations that varied from less than 1 km from a crop carrot field to more than 2.5 km from a crop carrot field. Carrot is a primarily outcrossing, insect pollinated species that is also capable of self-fertilization [49]; seed can be dispersed by both wind and animals [50,51]. This strategy was carried out in two regions of the United States: Nantucket Island (Nantucket County) in Massachusetts in the eastern United States where carrot is grown as a root crop and on the Olympic Peninsula in the Sequim-Dungeness Valley (Clallam County) in Washington State in the western United States where crop carrot is grown for seed production. Three populations (Cliff Rd, Polpis Rd, Tuckernuck: 45 individuals) located away from crop fields and two populations (Bartlett Farms, Moors End Farm: 84 individuals) located nearby to crop fields of wild carrot were sampled from Nantucket in Massachusetts. Three populations (Hemlock, Kendall, Medsker: 43 individuals) located away from crop fields and four populations (Eberle, Fasola, Fencebird, Prince: 93 individuals) located near crop fields of wild carrot were sampled from the Olympic Peninsula/Sequim-Dungeness Valley of Washington State. The majority of leaves and seeds were collected from publicly available land, no specific permissions or permits were required for these locations, and this study did not involve endangered or protected species. Two populations were collected from private land and specific permission was obtained by JRM from the landowners for collection. Note that germination rates of *Daucus* seed were generally high (> 90%), and seeds were allowed to grow for up to 8 weeks before tissue was collected for DNA purification.

The four carrot cultivars (total 19 individuals) that are grown as a root crop on Nantucket were obtained from Johnny’s Seed Company and Harris Seed Company (information kindly provided by the farmers on Nantucket). These varieties included ‘Purple Haze F1’, ‘Nelson F1’, ‘Nantindo F1’and open-pollinated ‘Scarlet Nantes’. It should be noted that even though carrot is cultivated for the root on Nantucket, farmers of both locations studied here report early bolters (plants flowering in their carrot fields in the first season) and plants occasionally flowering in their fields after harvest the following season (presumably missed during harvest the previous season). The three open-pollinated cultivars (total 49 individuals) grown in the Olympic Peninsula/Sequim-Dungeness Valley are self-selected lines grown by the farms: OP-1, OP-2, OP-3 (seed kindly provided by the farmers of the Olympic Peninsula). Since crop lines are continuously rotated (pers. comm. farmers of Nantucket and Olympic Peninsula/Sequim-Dungeness Valley), these lines were grouped into one population (per region) for the analyses presented here. Also note that carrot growers who grew a crop from the seed sources above, in both Nantucket and the Olympic Peninsula, reported no incidence of wild carrot outcrosses in their seed stocks.

Genomic DNA was isolated from a 2 cm portion of leaf tissue obtained from individual leaves using the SQ Omega Biotek Plant Kit (Atlanta, GA, USA) with the addition of 1% PVP and 1% Ascorbic Acid. DNA was quantified and quality assayed using a Nanodrop spectrophotometer (Thermo Scientific, MA). If DNA quality were low, samples were purified using an Omega Biotek E.Z.N.A. Cycle Pure Kit following the manufacturers protocol.

### Chloroplast DNA length variation and nuclear microsatellites

Length variation of the region between the trnG and trnS genes (S/G region) of the chloroplast genome was used as a chloroplast genetic marker. PCR amplification of this region was performed using the ‘S’ and ‘G’ primers from Hamilton [52]. The PCR was carried out in a total reaction volume of 15 μl, consisting of 1.5 μl of 10X PCR buffer (100mM KCl, 100mM Tris HCl (pH9.0), 80mM (NH4)2SO4, and 1.0% Triton X-100), 0.35 μl MgCl2 (25mM), 0.2 μl dNTPs (each at 20mM), 0.2 μl forward primer (5μM), 0.2 μl reverse primer (20μM), 0.2 μl fluorescently labeled M13 primer (following the methods of [53] as in [54]) and (10μM), 0.5 μl of Taq DNA polymerase, and 1 μl template DNA (20 ng/μl). The PCR conditions were: 3 min at 95°C; 10 cycles of 30 s at 94°C, 30 s at 65°C and 45 s at 72°C, annealing temperature decreasing to 55°C by 1°C per cycle, followed by 30 cycles of 30 s at 94°C, 30 s at 55°C, 45 s at 72°C, followed by 10 min at 72°C. The PCR amplicons were diluted 1:10 and visualized using an ABI 3130xl DNA sequencer (Applied Biosystems, Foster City, CA) with GeneScan 500 LIZ dye Size Standard (Applied Biosystems) included in each lane to allow for accurate fragment size determination. Alleles were scored using the software package GeneMarker v. 2.6.3 (SoftGenetics, State College, PA). Heteroplasmic individuals were characterized on the basis of more than one allele signal for a single individual following the methods of Ellis et al. [55].

A total of 15 nuclear Simple Sequence Repeat (SSRs or microsatellite) markers previously developed by [56] were used in this study: GSSRs 3, 4, 6, 7, 9, 11, 16, 24, 31, 35, 57, 65, 85, 107, and 111. These markers are scattered throughout the genome and include all nine linkage groups. Methods for PCR amplification and visualization follow as above for chloroplast DNA with a few adjustments in amounts of reagents (see S1 Table for details).

### Population genetic analyses

Measures of genetic diversity and genetic distance were assayed separately for the two regions studied here using the software GenAlEx v. 6.5 [57] for the two genomes. Given that heteroplasmy exists in wild populations of Queen Anne’s Lace and the mixture is usually skewed toward one haplotype/allele [43], we chose to score chloroplast markers on the basis of the major haplotype (essentially ignoring heteroplasmy in measures of genetic diversity and gene flow here). Nei’s [58] unbiased nuclear gene diversity uHe, chloroplast unbiased diversity cpuH, and the Shannon information index H were calculated for each population. Population structure was assessed using analysis of molecular variation (AMOVA; [59]), as implemented in GenAlEx, to hierarchically partition genetic variation and estimate Wright’s F_ST_ [60]. Statistical significance (i.e., H_0_ = no genetic differentiation among the populations) was determined by performing 1000 permutations. Further, genetic distance amongst individuals was investigated graphically using principal coordinates (PCO) analysis. For this, a standard genetic distance matrix [58] was constructed based on the nuclear multi-locus genotypes, the resulting matrix was used for the PCO analysis, and the first two principal coordinates were graphed in 2-dimensional space.

Population structure based on nuclear loci in each region (“Nantucket” and “Olympic”) was also investigated using the Bayesian, model-based clustering algorithm implemented in the software package *structure* [61]. For these analyses, individuals were assigned to K population genetic clusters based on their nuclear multilocus genotypes. The software, *structure*, assembles clusters by minimizing intra-cluster Hardy–Weinberg and linkage disequilibrium and, for each individual, the proportion of membership in a given cluster is estimated. Our analysis here did not use prior population information (i.e., USEPOPINFO was turned off). For each analysis, K = 1–10 population genetic clusters were evaluated with 5 runs per K value. For each run, the initial burn-in period was set to 200,000 with 500,000 MCMC iterations. The optimal number of K clusters was estimated using the delta K method of Evanno et al. [62] as implemented in Structure Harvester on the web [63]. When an optimal K was determined for either region, membership coefficients in each cluster were aligned across replicate runs using *CLUMPP* [64] using the *Greedy* algorithm. Finally, the cluster membership was visualized using the software *distruct* [65]. Pie charts of structure results were also graphically visualized using ArcGIS (Release 10.2.2. Redlands, CA: Environmental Systems Research Institute).

In order to estimate contemporary rates of gene flow among populations within regions and as a complement to our historical methods using traditional population genetic analyses, we used the software BayesAss 3.0.4 [66]. As advised by the authors, we varied seed numbers and monitored consecutive runs in order to diagnose convergence. Also, following the authors’ suggestion, delta values were adjusted so that the accepted numbers of changes were between 20–60%. MCMC runs were performed with 10,000,000 iterations after a burn-in of 1,000,000 and a sampling frequency of 1,000. The migration rates for each population, including the standard deviation of the marginal posterior distribution for each migration rate estimate, were recorded.

### Comparison of traditional population genetic measures versus ecological measures

Traditional population genetic measures of Nei’s [58] unbiased gene diversity (uHe) and Wright’s F_ST_ [60] were compared to those derived from Shannon information theory (i.e., from Ecology: Shannon diversity indices) [44–46]. The population genetic measures uHe and F_ST_ both measure aspects of genetic diversity and differentiation within and among populations. The measure uHe is the expected heterozygosity, or gene diversity, corrected for sample size for a given population and ranges from 0 to 1 with higher values indicating greater population genetic diversity. The measure F_ST_ describes the degree of genetic differentiation among populations and also ranges from 0 to 1; when F_ST_ is lower, populations do not differ greatly in terms of allelic diversity, and when F_ST_ is higher, populations are described as more genetically different from one another. The Shannon measures, Shannon’s H and S_HUA_ are analogous to the population genetic measures uHe and F_ST_ respectively: Shannon’s H measures levels of genetic diversity within a population but is not bounded by 1; S_HUA_ (also not bounded by 1) similarly measures the degree of genetic differentiation amongst sampled populations. For measures of uHe and Shannon’s H and F_ST_ and S_HUA_, populations were grouped according to their proximity to crop fields (see description above of collection strategy). A two-factor ANOVA was performed in JMP (v. 9) using crop proximity and locus as factors to investigate significance among diversity measures. A sign test (in JMP) was also performed to assay significance.

## Results

### Chloroplast DNA

**Fig 1:**
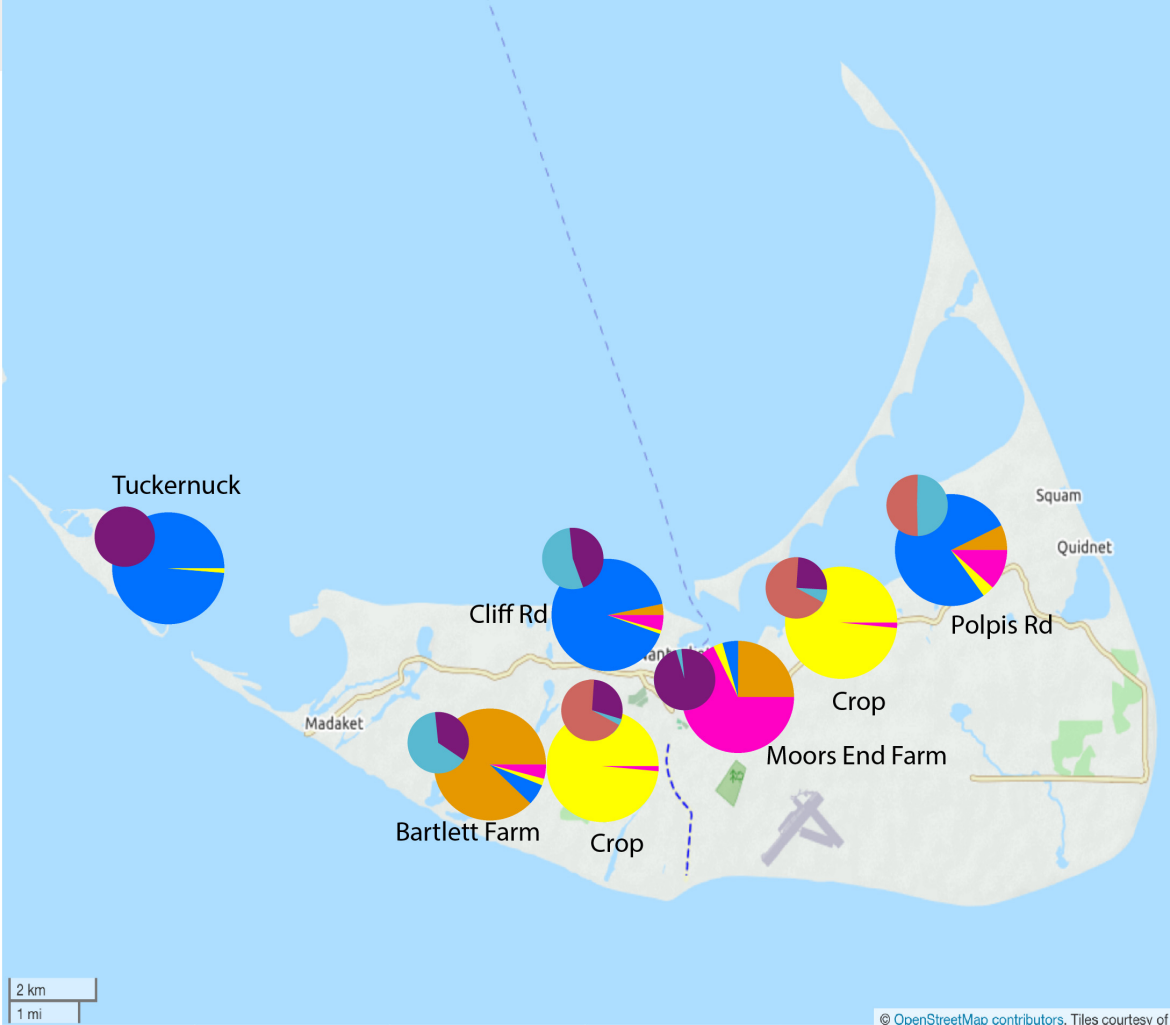
Nantucket *structure* analysis. Results of *structure* analysis for K = 4 clusters (large pie charts) and chloroplast haplotypic diversity (small pie charts) for Nantucket Island.

In Nantucket (the crop production sampling area), three haplotypes were recorded across the six populations (no private haplotypes). All populations except Tuckernuck were polymorphic for the chloroplast marker, and unbiased diversity ranged from 0 at Tuckernuck to 0.56 at Polpis Rd demonstrating substantial haplotypic variation across the island (Table 1). The F_ST_ value as estimated from AMOVA was 0.43 (p < 0.001) with haplotypic frequency showing some degree of structuring across populations (Fig 1). Two wild individuals (out of 129) from different populations showed evidence of chloroplast heteroplasmy, and four crop samples (out of 19) showed evidence of chloroplast heteroplasmy.

**Table 1:** Carrot population information and genetic diversity measures for nuclear and chloroplast loci.

Region	Population	Proximity	Number	uHe (SE)	Shannon H	cpuH	
**Nantucket**	Crop	—	19	0.59 (0.04)	1.09 (0.10)	0.50	
**Nantucket**	Cliff Rd	Away	19	0.60 (0.06)	1.16 (0.12)	0.53
**Nantucket**	Polpis Rd	Away	10	0.64 (0.05)	1.13 (0.11)	0.56	
**Nantucket**	Tuckernuck	Away	16	0.58 (0.03)	0.99 (0.06)	0.00	
**Nantucket**	Bart Farm	Near	37	0.73 (0.03)	1.56 (0.11)	0.47	
**Nantucket**	Moors Farm	Near	47	0.72 (0.04)	1.60 (0.12)	0.04	
**Olympic**	Crop	—	49	0.42 (0.06)	0.77 (0.12)	0.31	
**Olympic**	Hemlock	Away	14	0.80 (0.03)	1.71 (0.09)	0.62	
**Olympic**	Kendall	Away	16	0.67 (0.03)	1.25 (0.09)	0.46	
**Olympic**	Medsker	Away	13	0.78 (0.03)	1.64 (0.13)	0.69	
**Olympic**	Eberle	Near	36	0.76 (0.03)	1.60 (0.10)	0.48	
**Olympic**	Fasola	Near	11	0.62 (0.06)	1.16 (0.12)	0.33	
**Olympic**	Fencebird	Near	31	0.70 (0.03)	1.44 (0.08)	0.40	
**Olympic**	Prince	Near	15	0.69 (0.03)	1.30 (0.07)	0.25	

uHe = Nei’s (1978) unbiased nuclear gene diversity ± standard error (SE). Shannon H = Shannon Information Index for nuclear loci (Peakall and Smouse 2012) ± standard error (SE). cpuH = chloroplast unbiased diversity (Peakall and Smouse 2012).

**Fig 2:**
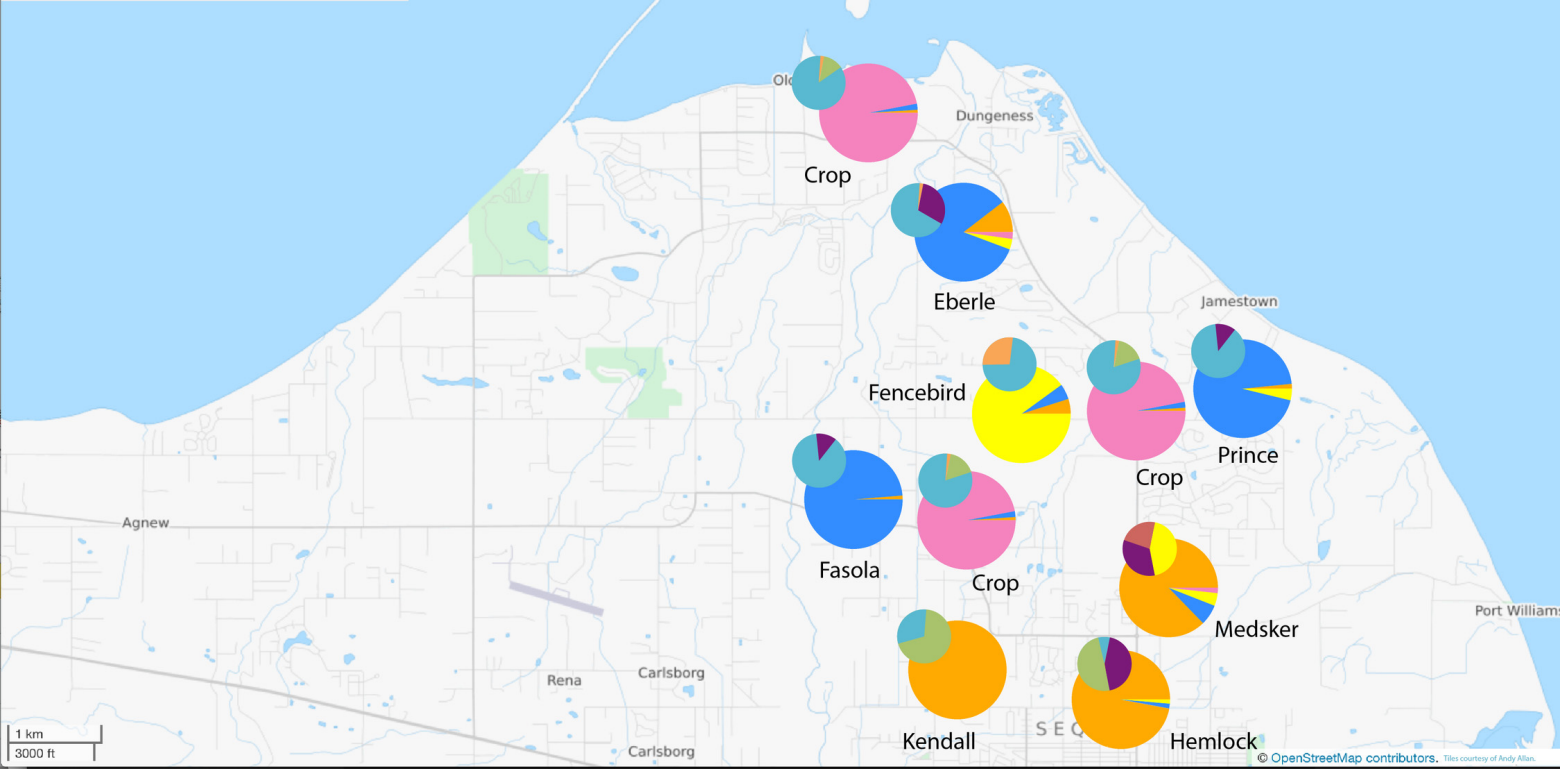
Olympic *structure* analysis. Results of *structure* analysis for K = 4 clusters (large pie charts) and chloroplast haplotypic diversity (small pie charts) for Olympic Peninsula/Sequim-Dungeness Valley.

In the Olympic Peninsula (the seed production sampling area), six haplotypes were recorded across the eight populations (no private haplotypes). All populations were polymorphic for the chloroplast marker and unbiased diversity ranged from 0.31 in the crops to 0.69 in the Medsker Rd population demonstrating substantial haplotypic variation across the region (Table 1). The F_ST_ value as estimated from AMOVA was lower than on Nantucket: 0.29 (p < 0.001) with haplotypic frequency showing some degree of structuring across populations with populations on the northern end of the valley harboring a different haplotype frequency than populations farther south and farther from crop fields (Fig 2). Thirty-four wild individuals (out of 136) from three populations showed evidence of chloroplast heteroplasmy, and one crop sample (out of 49) showed evidence of chloroplast heteroplasmy.

### Nuclear microsatellites

**Fig 3:**
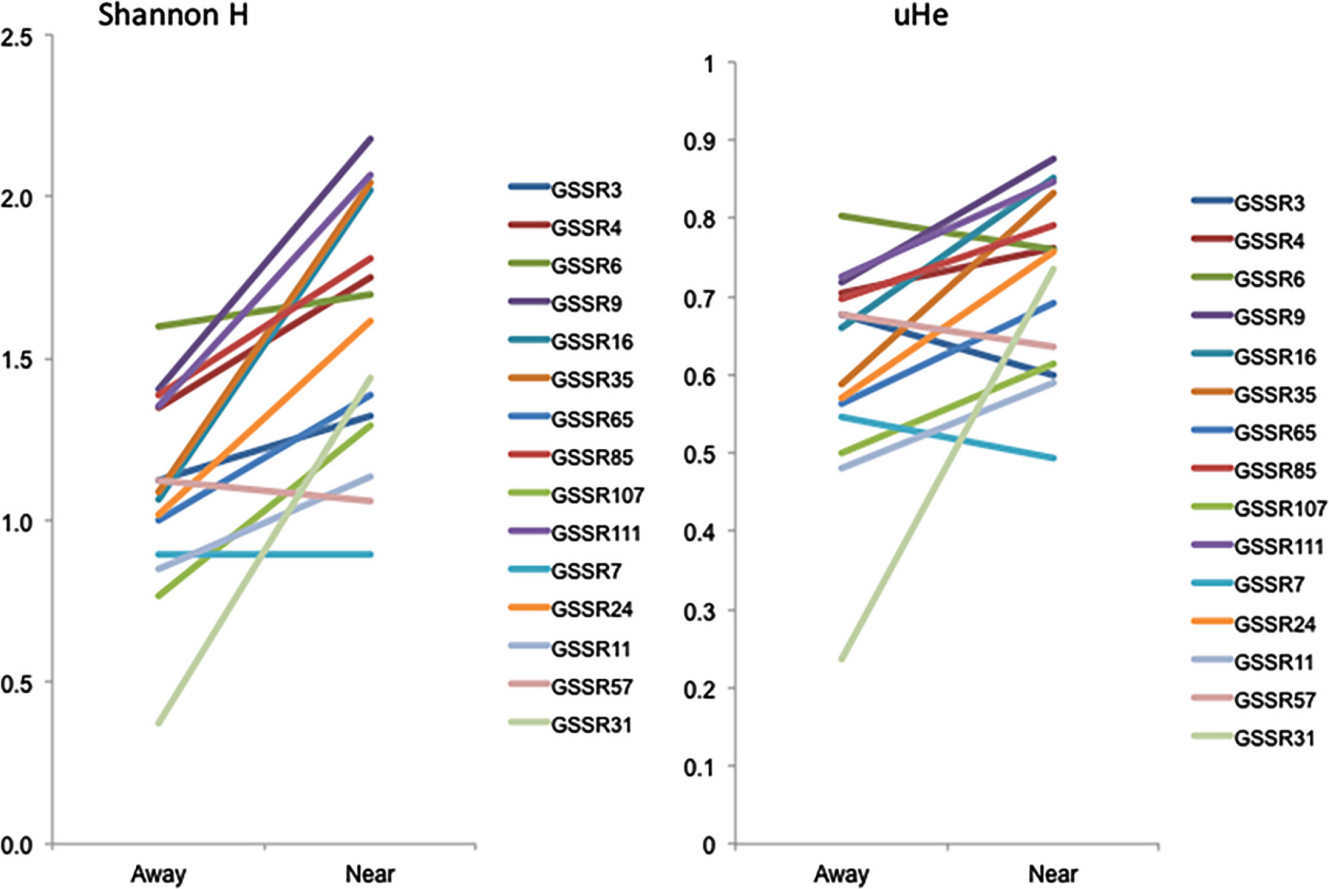
Nantucket Shannon H and gene diversity. Comparison of Shannon H and unbiased gene diversity on a per locus basis for away versus near wild carrot populations for Nantucket.

In Nantucket (the crop production sampling area), all fifteen nuclear SSRs were polymorphic in all populations, and unbiased multilocus gene diversity ranged from 0.58 on Tuckernuck to 0.73 at Bartlett Farms (Table 1). Multilocus Shannon information index values (Shannon H) varied from 0.99 on Tuckernuck to 1.60 at Moors End Farm (Table 1). Single locus values were also calculated for both uHe and Shannon H and compared using a sign test and proximity and locus two-factor ANOVA. For both metrics, wild populations in close proximity to crop fields showed overall *higher* single locus values of uHe and Shannon H, though the Shannon H measures appeared more sensitive to these differences (uHe: 11/15 loci higher in near versus away populations; sign test *p* = 0.059; proximity factor ANOVA, *F* = 9.01, *p* = 0.0095 and Shannon H: 14/15 loci higher in near versus away populations; sign test *p* < 0.0001; proximity factor ANOVA, *F* = 28.44, *p* < 0.0001; Fig 3).

**Fig 4:**
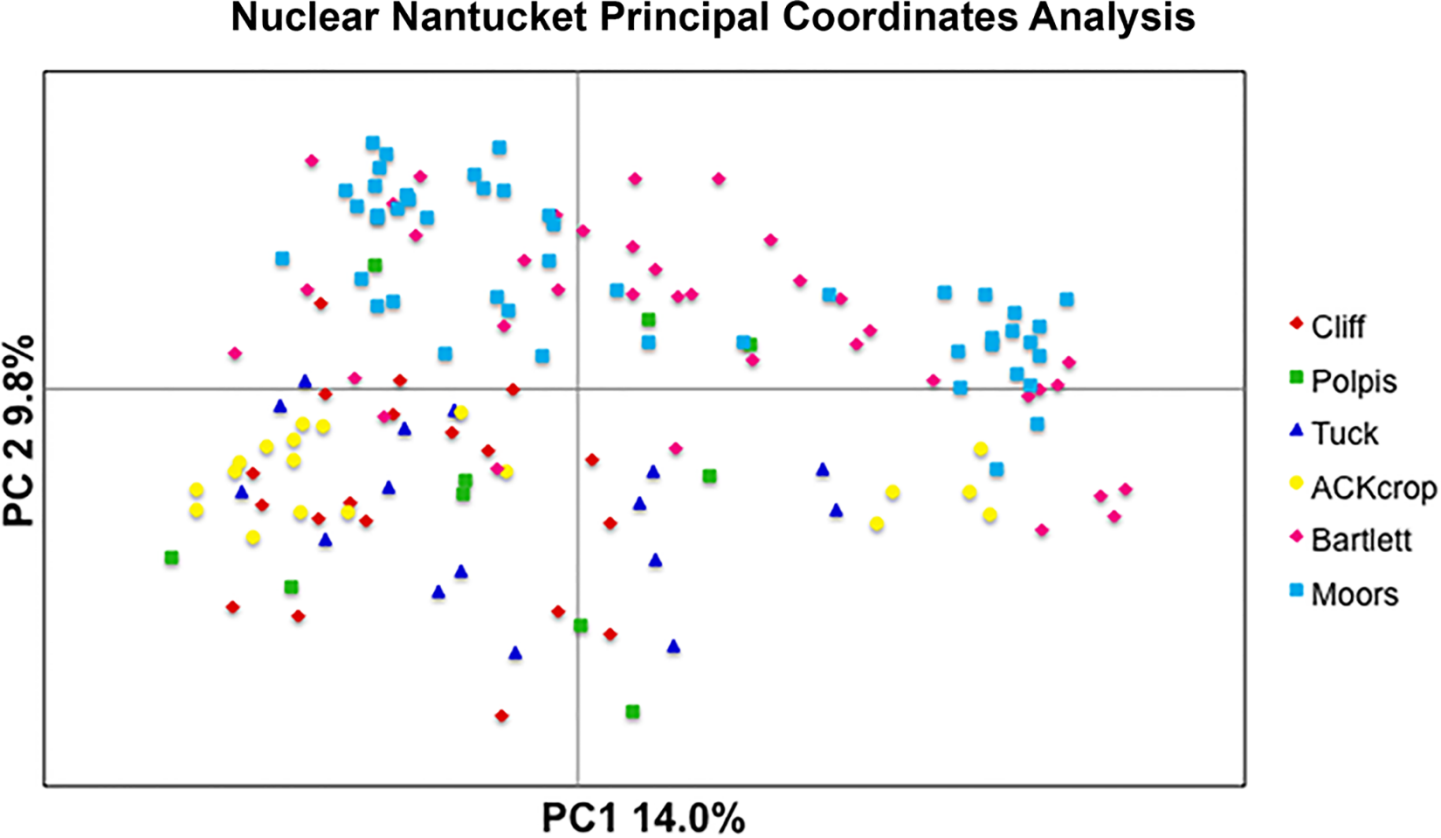
Nantucket Principal Coordinates Analysis. Results of the nuclear Nantucket Principal Coordinates Analysis graphically represented in 2-dimensional space.

In terms of population structure, the multilocus F_ST_ value as estimated from AMOVA was 0.14 (p < 0.001) with paired population F_ST_ values ranging from 0.11 (Polpis Rd to Cliff Rd—both away populations) to 0.30 (Crops to Cliff Rd) (S1 Fig). The 2-dimensional graphical representation of the populations (Fig 4) demonstrates a likely high degree of population connectivity across the island with some structure but substantial overlap amongst populations. The DeltaK method of Evanno et al. [62] as applied to the Bayesian clustering method *structure* [61] provided support for the presence of four genetically distinct clusters in Nantucket (Fig 1 and S2 Fig). These clusters roughly corresponded to the crop individuals, away proximal populations and then the two separate wild populations located near carrot farms; yet some degree of genotypic/allelic overlap can be seen among individuals (S2 Fig).

When F_ST_ was calculated on the grouping of populations based on proximity in Nantucket (the crop production sampling area), the near to crop populations versus far from crop populations demonstrated slightly lower values: away wilds to crops F_ST_ = 0.23, near wilds to crops F_ST_ = 0.21. A single locus approach based on proximity also failed to demonstrate any significant differences in away versus near measures of F_ST_, proximity and locus two-factor ANOVA *p* = NS. However, when Shannon diversity indices were used, the difference between distance measures was greater: away wilds to crops Shannon Hua = 0.21; near wilds to crops Shannon Hua = 0.12, and the single locus approach demonstrated that the Shannon Hua values were lower in near versus away populations, proximity and locus two-factor ANOVA, F = 25.12 *p* = 0.0002. Contemporary estimates of migration using the software program BayesAss yielded estimates of migration rates (range: 0.068% to 21%) that were relatively similar across populations for Nantucket. The largest rate was the Cliff Rd population as an immigration source for the Polpis Rd population—both populations located away from crop fields (S2 Table).

**Fig 5:**
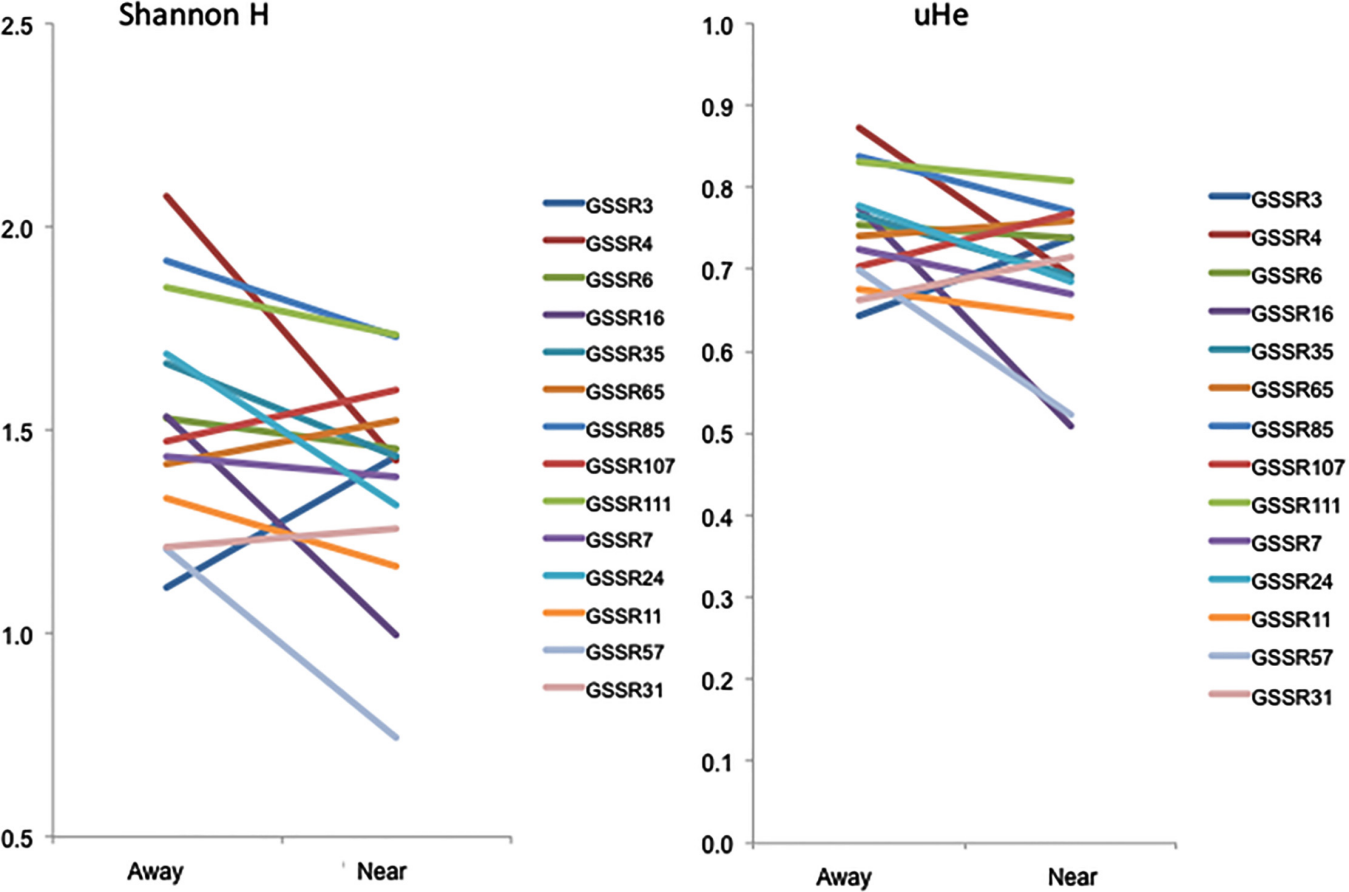
Olympic Shannon H and gene diversity. Comparison of Shannon H and unbiased gene diversity on a per locus basis for away versus near wild carrot populations for Olympic.

In the Olympic Peninsula (the seed production sampling area), fourteen nuclear SSRs (GSSR9 yielded unreliable amplification and was removed from further analyses) were polymorphic in all populations, and unbiased multilocus gene diversity ranged from 0.42 in the crops to 0.80 at Hemlock Rd population (Table 1). Multilocus Shannon information index values (Shannon H) varied from 0.77 in the crops to 1.71 at Hemlock Rd population (Table 1). Single locus values were also calculated for both uHe and Shannon H and compared using a sign test and proximity and locus two-factor ANOVA. Different from Nantucket, for both metrics, wild populations in close proximity to crop fields showed overall *lower* single locus values of uHe and Shannon H, and the Shannon H measures appeared more sensitive to these differences (uHe: 11/15 loci *lower* in near versus away populations; sign test *p* = NS; proximity factor ANOVA *p* = NS and Shannon H: 11/15 loci *lower* in near versus away populations; sign test *p* = NS; ANOVA, proximity factor *F* = 4.76, *p* = 0.027; Fig 5).

**Fig 6:**
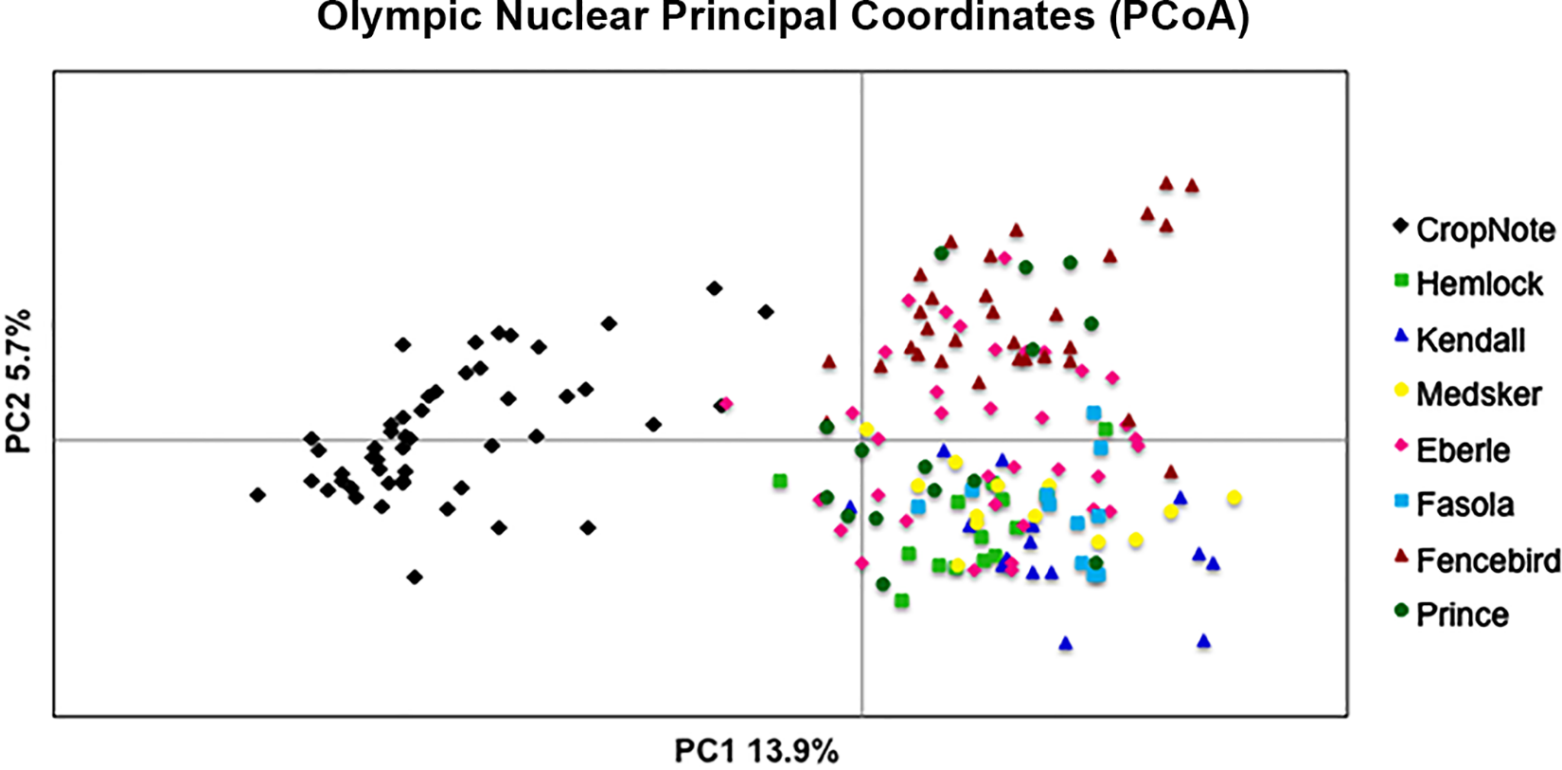
Olympic Principal Coordinates Analysis. Results of the nuclear Olympic Principal Coordinates Analysis graphically represented in 2-dimensional space.

In terms of population structure, the multilocus F_ST_ value as estimated from AMOVA was 0.17 (p < 0.001) with paired population F_ST_ values ranging from 0.049 (Hemlock to Medsker—both away populations) to 0.48 (Crops to Fasola—a near population) (S3 Fig). The 2-dimensional graphical representation of the populations (Fig 6) demonstrates a substantial amount of population connectivity across the valley with some structure but substantial overlap amongst populations. The DeltaK method of Evanno et al. [62] as applied to the Bayesian clustering method *structure* [61] provided support for the presence of four genetically distinct clusters in Olympic (Fig 2 and S4 Fig). These clusters corresponded to the crop individuals, away populations, and near to crop field populations. Still some degree of genotypic/allelic overlap within individuals can be seen (S4 Fig).

When F_ST_ was calculated on the grouping of populations based on proximity in Olympic (the seed production sampling area), the near to crop populations versus away to crop populations demonstrated slightly lower values: away wilds to crops F_ST_ = 0.27, near wilds to crops F_ST_ = 0.22. The single locus approach based on proximity also failed to demonstrate any significant differences in away versus near measures of F_ST_, proximity and locus two-factor ANOVA (*p* = NS). When Shannon diversity indices were used, the away populations also showed greater divergence than the near populations but not as large as the difference in Nantucket: away wilds to crops Shannon Hua = 0.22; near wilds to crops Shannon Hua = 0.17; yet still the single locus approach demonstrated that the divergence measures of Shannon Hua were lower in near versus away populations, proximity and locus two-factor ANOVA, F = 20.18 *p* = 0.0006. Contemporary estimates of migration using the software program BayesAss yielded estimates of migration rates (range: 0.058% to 21%) that were relatively similar across populations for Olympic. The largest rate was the Eberle population as an immigration source for the Fasola population—both populations located near crop fields (S2 Table). Raw data allele scores for nuclear and chloroplast loci are located in S3 Table.

## Discussion

### Patterns of nuclear gene flow and comparison of methods

A major goal of this study was to investigate the evidence for gene flow among populations of carrot with particular interest in levels and patterns of gene flow occurring amongst crop and wild populations. Thus, we sampled from wild carrot locales that were within 1km of a crop field and locations that were more than 2.5km away from crop fields in two regions where carrot is grown in the United States (recognizing that this represents a reasonable estimate of historical levels of gene flow). Using both methods that detect historical levels of gene flow (F_ST_) as well as methods more traditionally used for assessing ecological species diversity (i.e., Shannon diversity indices), the results presented here demonstrate a significant level of gene flow among populations both on Nantucket and in the Olympic Peninsula/Sequim-Dungeness Valley. Our results show that while populations do exhibit genetic structuring, some degree of population connectivity among crop and wild carrot populations certainly occurs. Moreover, gene flow appears to occur at a significant rate with respect to *both* nuclear and cytoplasmic genomes. The measures of nuclear F_ST_ reported here (Nantucket F_ST_ = 0.14; Olympic F_ST_ = 0.17) are on par with those of other angiosperm species as reported by Petit et al. [37] where the biparental mean ± standard error/SE from 77 studies was 0.184 ± 0.002. Additionally, a recent study including crop and a global sampling of wild carrot individuals reported moderate levels of genetic differentiation (0.19; [27]).

When we compared measures of genetic differentiation here grouping populations based on their proximity to crop fields, we found that in both regions (Nantucket and Olympic) populations that were closer to crop fields harbored lower levels of differentiation as compared to populations that were farther. This finding held when using both measures of F_ST_ and Shannon diversity indices; however, the Shannon measures were more sensitive to these differences (stronger statistical support). This result aligns with recent suggestions by several authors to make use of both types of measures when investigating population structure and gene flow [44–46] especially between crop and wild species populations [47]. Moreover, Campbell et al. [47] suggest taking a single locus based approach (analyzing single loci separately along with total multilocus measures). When we followed this approach, we found that accounting for single locus effects yielded more sensitivity and stronger statistical support for differentiation and levels of genetic diversity for Shannon diversity indices but not for conventional measures of F_ST_ (similar to the findings of [47]).

An additional finding of Campbell et al. [47] was that putative hybrid populations (between wild plants and their outcrossing crop relatives) demonstrated increased genetic diversity (using ecological measures) when compared to non-hybrid populations. Given this, one might predict that our populations in close proximity to crop fields would show evidence of increased genetic variation. Interestingly, this increased variation was the case for the Nantucket region but the opposite trend was found in the Olympic region (with Shannon measures again being more sensitive than those of unbiased gene diversity). We can currently only speculate as to why these differences between regions might occur: one possibility is the difference in management strategies of wild carrot populations between the two regions. One possibility relates to the levels of genetic variation in the crops: Nantucket crops were more genetically diverse in all measures when compared to crops on the Olympic Peninsula. One finding to note here is that given the Nantucket region is a root field site, we might have expected little to no gene flow at this location between crop and wild populations; however, our study did find population connectivity at the genetic level though this varied depending on the marker type used (measures of population structure at nuclear loci were similar in both regions while chloroplast population structure was higher on Nantucket).

In both regions, wild carrot is very common and on Nantucket it is regarded as an attractive wild flower that residents cultivate in their own home gardens. The species is also tolerated or ignored on the farm properties where crop carrot is grown with farmers occasionally reporting early bolters and plants with intermediate root morphology (potential F_1_ hybrids) providing evidence for outcrossing with wild carrot when seed for that crop was produced. The situation is quite different in the Olympic Peninsula/Sequim-Dungeness Valley where commercial carrot seed production is an important priority of the region. Both the local farmers and the Clallam County Extension Office have active campaigns against wild carrot due to concerns of contamination of crop seed. “Wanted” posters can be found in the area stating “Queen Anne’s Lace, Wanted Dead”, and the community is advised to cut the umbels of the plants off before they set seed.

Another possibility is in the difference in carrot seed sources used by farmers in the two regions. Farmers in the Nantucket region are often using hybrid carrot seed and likely very little of that carrot seed has been produced locally. So any seed set in nearby wild carrot populations that results from pollination with cultivated carrot is relatively rare. And with minimal wild carrot control, those pollinations more likely involved wild carrots closer to crop production fields. In contrast, production of open-pollinated carrot seed is an ongoing practice by farmers in the Olympic Peninsula. While wild carrot control measures are applied in the Olympic Peninsula, the ongoing annual presence of pollen from cultivated carrots may well have resulted in gene flow to wild carrots from local seed production fields on a regular basis to the extent that wild carrots closer to production fields are more similar to locally cultivated carrots that more distant populations of wild carrots. Additional surveys of populations near and far from crop fields in both regions would be useful to see if this pattern holds with a broader sampling. Furthermore, an evaluation of specific alleles associated with domestication traits like root color, lateral root formation, and early bolting (floral initiation) in populations near to and far from crop production fields in future studies may reflect differential selection for those genomic regions relative to random genetic markers.

### Patterns of chloroplast gene flow

While measures of nuclear genetic differentiation follow those of other angiosperm species, the measures of population structure based upon the chloroplast marker were much lower than typically found in angiosperm plant populations (Nantucket F_ST_ = 0.43; Olympic F_ST_ = 0.29). Petit et al. [37] summarized 124 angiosperm population genetic studies that surveyed maternally inherited markers and found that the mean genetic differentiation was 0.637 (± 0.002 standard error). The median value was 0.646 with the 25^th^ and 75^th^ quartiles being 0.416 and 0.871 respectively. Our chloroplast differentiation values were either on the extreme low end (for Nantucket) or well outside the range of the quartiles (for Olympic). Other studies have found similar low values for carrot organellar population structure. Ronfort et al. [67] examined population structure in populations of wild carrot in France and found levels of mitochondrial genetic differentiation to be extremely low (0.08); while Mandel et al. [40] also found lower than expected values of mitochondrial population structure in natural populations of wild carrot in the Eastern United States (0.34).

Several explanations can be put forward for this finding of lower than expected cytoplasmic gene flow in our study. One possibility is that seeds of carrot are better dispersers (i.e., potential for greater gene flow) than many other angiosperm species such that the chloroplast genome shows significantly lower levels for differentiation as compared to other taxa [37]. Indeed, carrot seeds do tend to move substantial distances in the wild being dispersed by both animals and wind with wind possibly contributing more to dispersal than animals [50,51]. Thus, it appears that efficient dispersal of seeds may play a role in increasing population connectivity and decreasing population structure with respect to organellar genomes. Furthermore, prior to the development of large-scale commercial carrot seed production in regions of the world with little wild carrot and accompanying management systems put in place to minimize the occurrence of outcrosses (both in just the last 75 years), carrot seed was produced locally in Europe, Asia, and the Americas where wild carrot is known to have been widespread. This opened up the likelihood that cytoplasmic gene flow has been ongoing between wild and cultivated carrot throughout most of the history of the crop, and perhaps accounts for the reduced genetic differentiation observed today.

The action of metapopulation dynamics and frequent extinction and recolonization events may also play a role in determining population genetic structure in carrot populations. Ronfort et al. [67] point out that since wild carrot often inhabits newly disturbed areas and can be quickly overturned by later successional species, populations may experience some of the effects associated with extinction-recolonization dynamics. In metapopulations, both the number of colonists (starting new populations) and migration (among current populations) can influence gene flow and population genetic differentiation. When the number of colonists is high relative to among population migration rates, extinction and recolonization can enhance gene flow and diminish population genetic differentiation [68,69]. Given this, aspects of carrot population dynamics could play a role in determining the degree of differentiation especially if seeds are efficient dispersers to new unoccupied patches or habitats. It should also be noted that in both regions where we sampled these carrot populations, Queen Anne’s Lace is virtually ubiquitous throughout Nantucket Island and the Olympic Peninsula/Sequim-Dungeness Valley (despite efforts to remove/control wild carrot here).

Another possibility is that the chloroplast genome is not transmitted strictly maternally but occasionally moves via pollen as well. Paternal leakage would allow chloroplast genes to move through both seed *and* pollen leading to higher levels of cpDNA gene flow and thus lower levels of population genetic differentiation. Occasional paternal inheritance of organellar DNA, sometimes termed “paternal leakage” has been documented in numerous plant species (nicely reviewed in [41]). Paternal inheritance of chloroplast DNA would tend to reduce measures of F_ST_ aligning them closer to F_ST_ values of the nuclear genome since the chloroplast genome could move in both seed and pollen. Paternal leakage of the chloroplast genome is also one factor that can lead to heteroplasmy within an individual (having a mixture of different organellar genes/genomes) making heteroplasmy an indirect indicator of paternal leakage in crosses [41]. Paternal inheritance of chloroplasts was previously reported in hybrid crosses between different species of *Daucus* [70]. And recently, heteroplasmy of the mitochondrial genome has been documented in both crop [71] and wild carrot [43]. In the current study, we found evidence for heteroplasmy of the chloroplast genome in Nantucket and to a greater degree in Olympic Peninsula. Further supporting the potential for paternal leakage to contribute to chloroplast gene flow here is the finding of greater heteroplasmy in Olympic accompanied by a lower level of population genetic differentiation there. Further study including broader population sampling and crossing studies are needed to determine which, or which combination, of these possibilities contributes to the substantial degree of chloroplast gene flow in carrot populations.

### Implications for transgene escape

Our findings have implications for crop-wild transgene escape especially that of organellar transgene escape. Two observations from the literature apply to this: 1) paternal leakage of otherwise maternally inherited cytoplasmic genomes is often associated with hybridization events [72–74]. For example, Boblenz et al. [70] found evidence for paternal inheritance of plastids in interspecific crosses among *Daucus* species. And 2) paternal transmission may also be enhanced in species that exhibit gynodioecious mating systems (consisting of both hermaphrodites and male-sterile females) as well as crop breeding programs that utilize cytoplasmic male sterility (CMS) like carrot [41,75]. The first observation that paternal leakage is enhanced in hybridization events suggests that divergence may play a role in permitting leakage in crosses such that it is more likely for leakage to occur in crosses between divergent populations (e.g., crop to wild). And secondly, species including crops that exhibit CMS may be particularly susceptible to paternal leakage of cytoplasmic DNA in their male-fertile lineages (note that CMS is essentially a breeding tool, and that male fertility is typically restored in seed sold to farmers), and thus cytoplasmic transgene escape. Therefore, studies which investigate heteroplasmy and paternal leakage in crop wild systems (especially those considering genetic modification) are certainly warranted. Finally, our study examined wild populations at distances less than, and greater than, 2.5km from crop fields. While we showed that gene flow is more abundant nearby to crop fields, gene flow likely occurs at greater distances as well. Certainly, consistent with previous findings [49] our study indicated that a minimum distance of 2.5km should be maintained, when possible, to limit the potential for gene flow with greater distances providing additional protection against gene flow to wild carrot populations.

## Supporting Information

S1 Fig Pairwise values of F_ST_ for Nantucket. (TIF)

S2 Fig Individual *structure* results for K = 4 clusters for Nantucket. (TIF)

S3 Fig Pairwise values of F_ST_ for Olympic. (TIF)

S4 Fig Individual *structure* results for K = 4 clusters for Olympic. (TIF)

S1 Table Details of PCR reactions for each locus. (XLSX)

S2 Table Migration estimates for each population for Nantucket and Olympic. (XLSX)

S3 Table Raw data microsatellite allele calls. (XLSX)
