# Supplementary figures and images for: Patterns of Gene Flow between Crop and Wild Carrot, Daucus carota (Apiaceae) in the United States
Source: PLoS One. 2016 Sep 7;11(9):e0161971. doi: 10.1371/journal.pone.0161971 (PMC5014312; doi:10.1371/journal.pone.0161971)

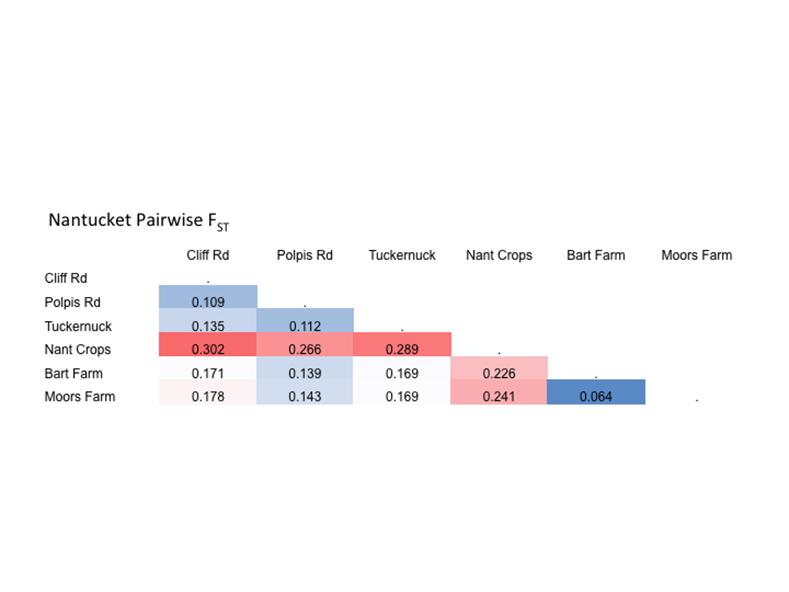

Supplement: S1 Fig — (TIF) [file pone.0161971.s001.tif]

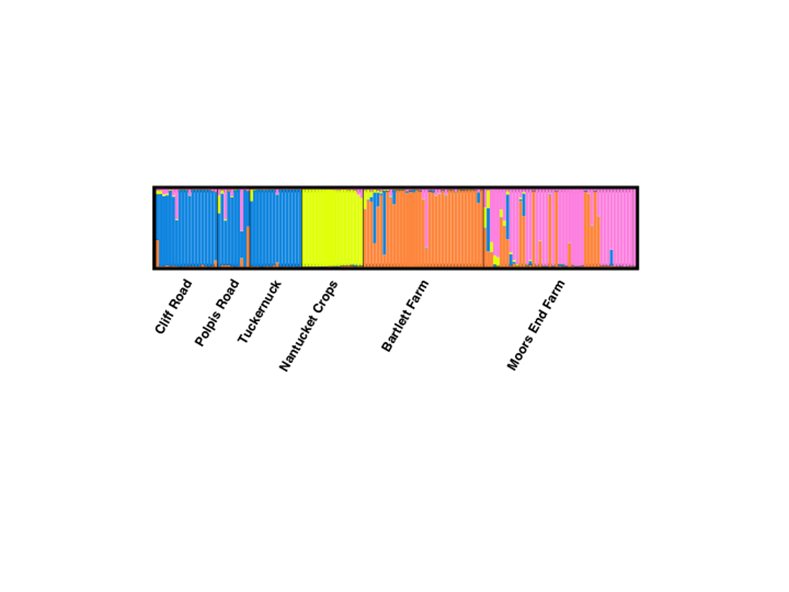

Supplement: S2 Fig — (TIF) [file pone.0161971.s002.tif]

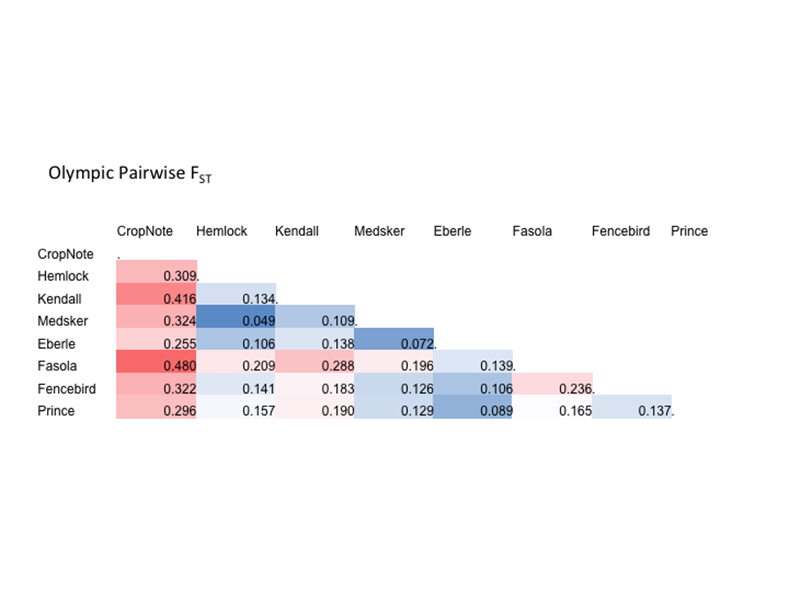

Supplement: S3 Fig — (TIF) [file pone.0161971.s003.tif]

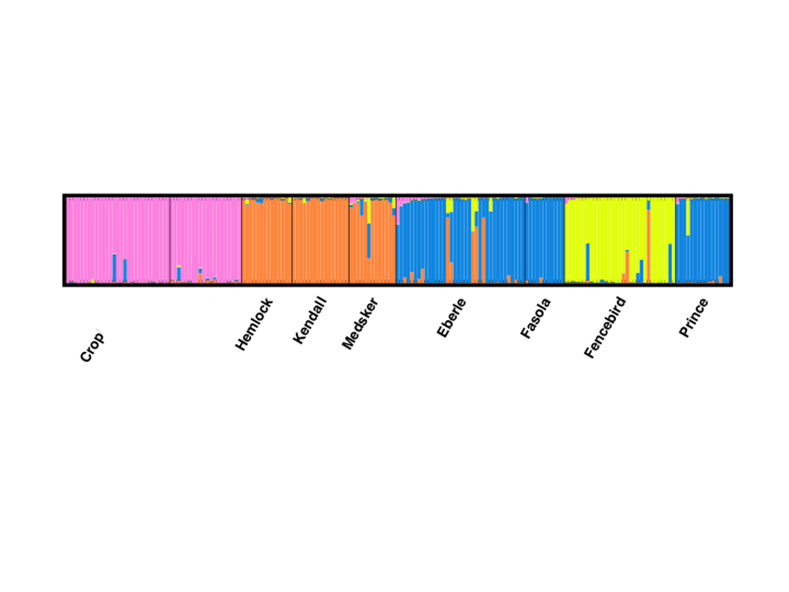

Supplement: S4 Fig — (TIF) [file pone.0161971.s004.tif]
